# Natural variation at the *Drosophila melanogaster Or22* odorant receptor locus is associated with changes in olfactory behaviour

**DOI:** 10.1098/rsob.210158

**Published:** 2021-09-29

**Authors:** Katherine H. Shaw, Craig I. Dent, Travis K. Johnson, Alisha Anderson, Marien de Bruyne, Coral G. Warr

**Affiliations:** ^1^ Tasmanian School of Medicine, University of Tasmania, Hobart 7000, Tasmania, Australia; ^2^ School of Biological Sciences, Monash University, Clayton 3800, Victoria, Australia; ^3^ Ecosystems Sciences, CSIRO, Black Mountain, Australian Capital Territory 2601, Australia; ^4^ School of Molecular Sciences, La Trobe University, Bundoora 3083, Victoria, Australia

**Keywords:** odorant receptor, *Drosophila melanogaster*, olfaction, behaviour, natural selection

## Abstract

In insects, many critical olfactory behaviours are mediated by the large odorant receptor (*Or*) gene family, which determines the response properties of different classes of olfactory receptor neurons (ORNs). While ORN responses are generally conserved within and between *Drosophila* species, variant alleles of the *D. melanogaster Or22* locus have previously been shown to alter the response profile of an ORN class called ab3A. These alleles show potential clinal variation, suggesting that selection is acting at this locus. Here, we investigated if the changes seen in ab3A responses lead to changes in olfactory-related behaviours. We show that variation at the *Or22* locus and in the ab3A neurons are not fully compensated for by other ORNs and lead to overall changes in antennal odorant detection. We further show that this correlates with differences in odorant preference behaviour and with differences in oviposition site preference, with flies that have the chimaeric short allele strongly preferring to oviposit on banana. These findings indicate that variation at the *Or22* locus leads to changes in olfactory-driven behaviours, and add support to the idea that the ab3A neurons are of especial importance to the ecology of *Drosophila* flies.

## Graphical abstract

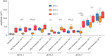

## 1. Introduction

Animals rely on their sense of smell to discriminate between odours in order to identify and locate mates, dangers and food sources. Flying insects need to be able to do all of this rapidly, and additionally use their chemosensory systems to identify the optimal places to lay their eggs. In insects, odour identity is combinatorially encoded through the inputs from a large number of classes of olfactory receptor neurons (ORNs) [1–3], whose responses are determined by the olfactory receptors they express [4–6]. One of the major families of olfactory receptors in insects is the rapidly evolving *Or* gene family, which encode highly divergent ligand-gated ion channels and primarily detect volatile odorants [7]. The odour responses of this family and their mapping to individual ORN classes have been best characterized in *Drosophila melanogaster*, where there are 62 Ors. In *D. melanogaster*, individual Ors/ORNs can be either broadly tuned (responding strongly to a wide range of odorants) or narrowly tuned (responding strongly to one or a few odorants), albeit with there being a continuum in the breadth of tuning rather than a strict dichotomy [8,9].

We previously reported one of the first described cases of within-species variation in responses of a *D. melanogaster* ORN, the ab3A neuron [10]. Among many ORN classes, this ORN is also one of only a few known to show interspecies variation between closely related *Drosophila* species [11,12]. The olfactory receptor locus that determines ab3A response is the *Or22* locus, and we showed that naturally occurring variation at this locus within *D. melanogaster* causes alterations to ab3A neuronal responses. Specifically, the *Or22* locus variant found in the common laboratory strain Canton S has two genes encoding Ors at this locus, *Or22a* and *Or22b* (the long allele [10,13]), however the Or22b protein is not functional. In these flies, Or22a produces an ab3A response phenotype we call ab3A-1. A major variant at this locus that has been found in wild populations [14–16] is a short allele in which a chimaeric receptor called Or22ab produces a different odour response profile that we call ab3A-2. We also identified a third phenotype with yet another different odour response profile (ab3A-3), in which there is still a long allele (with two gene copies) but *Or22a* is not expressed and instead a functional version of Or22b determines ab3A response [10]. In general, ab3A-1 neurons respond most strongly to the larger esters tested (e.g. ethyl hexanoate, methyl hexanoate and ethyl octanoate), ab3A-2 neurons to small acetate esters (e.g. propyl acetate, butyl acetate and pentyl acetate) and ab3A-3 neurons to smaller ethyl esters (e.g. ethyl propionate, ethyl butanoate and ethyl 2-methylbutanoate).

Frequencies of the long and short alleles at this locus have been proposed to be clinally varying in Australia, with the long allele being fully penetrant in the south and the short allele appearing at high frequency in the north [14]. Clinal variation can indicate that selection is acting at a locus [17,18]. For selection to be acting at an odorant receptor locus we might expect that the variation in neuron responses caused by the genetic variants has behavioural consequences. In support of this possibility, the ab3A neuron has already been implicated as an important modifier for host-preference behaviour in *D. melanogaster* and other *Drosophila* species [16,19–21]. Moreover, the *Or22ab* allele has been associated with altered host-seeking behaviour in a wild *D. melanogaster* population [16]. However, it is also possible that the observed clinal allele pattern in Australia is due instead to the *Or22* locus being in linkage disequilibrium with another locus of ecological importance.

Here, we therefore investigated if the different *Or22* variants cause changes in behaviours that may be important for individual fitness. We first show that broader antennal neuronal responses to odours are impacted by ab3A response variation. We further provide evidence of an association between ab3A phenotype and behavioural responses in two different olfactory behaviour paradigms. Taken together, our data strongly suggest that variation at the *Or22* locus leads to changes in overall odorant detection by the antenna that are associated with changes in biologically significant behaviours.

## 2. Results

### 2.1. Variation in ab3A phenotype alters overall antennal detection of ab3A ligands

The ab3A neuron is broadly tuned, with significant responses (greater than 50 spikes s^−1^) described to at least 50 odorants [9]. The sensitivity of this neuron to different odorants varies among different strains [10,16], and the neuron is, like most ORNs, more specific in the odorants it responds to at lower odorant concentrations [22]. Most, if not all, of the odorants detected by ab3A neurons are also detected by other ORNs, for example, pentyl acetate is also detected by ab1A, ab5B, ab6A and ab7A neurons, and ethyl butanoate is also detected by ab1A, ab8A, ab8B, ab10A, ab3B and ab2B neurons [1,9]. We therefore wondered if we would be able to detect differences in response between strain with the different ab3A phenotypes at the level of large neuronal populations, or if perhaps some compensatory changes might occur. We measured responses from populations of neurons across the antenna using electroantennogram recordings (EAGs). Rather than measuring the response of a single neuron, an EAG signal represents the summed responses of all ORNs in the vicinity of the recording electrode [23] (see [1,24] for diagram of ORN distribution on the antenna).

**Figure 1.**
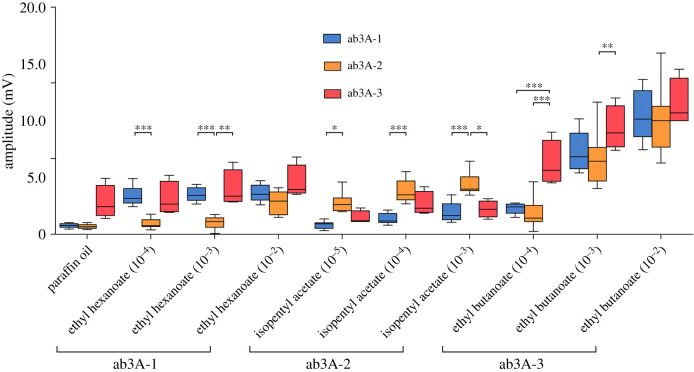
Flies with different ab3A phenotypes show variation in EAG responses. Isogenic lines with the three different ab3A phenotypes show differences in EAG amplitudes for lower concentrations of relevant odorants (2-way ANOVA with Bonferroni post-tests, *p < 0.05, **p < 0.01, ***p < 0.001). Paraffin oil is the solvent control, doses were selected as lowest dose giving maximum response (10) ±1 dose. Data presented as median + interquartile ranges; *n* = 9 for ab3A-1 and ab3A-2, *n* = 4 for ab3A-3.

We tested the EAG responses of fly lines isogenic (fully homozygous) for the second chromosome that individually exhibit the ab3A-1, ab3A-2, and ab3A-3 phenotypes to serial dilutions of odorants that we previously showed were able to distinguish between the three phenotypes at the single neuron level [10]. Odorant concentrations were selected based on our previous data on the ab3A responses to serial dilutions of these odorants, and we selected the lowest dose that gave the maximum response from the relevant phenotype and one dose either side of this. For ethyl hexanoate and ethyl butanoate, this gave a range from 10^−4^ to 10^−2^, while for isopentyl acetate this gave a range from 10^−5^ to 10^−3^. Paraffin oil was also tested as the solvent control. We found significant differences in EAG responses between flies with the different ab3A phenotypes (figure 1; electronic supplementary material, table S1). Flies with the ab3A-1 phenotype show significantly higher EAG responses to the ab3A-1 ligand ethyl hexanoate at 10^−4^ and 10^−3^ than do ab3A-2 flies, but not ab3A-3 flies. ab3A-2 flies show significantly higher responses to the ab3A-2 ligand isopentyl acetate than ab3A-1 flies at all tested doses, and than ab3A-3 flies at the 10^−3^ dose. ab3A-3 flies show higher responses to its ligand, ethyl butanoate, than both ab3A-1 and ab3A-2 flies at 10^−4^, and ab3A-2 flies at 10^−3^. We note that all three phenotypes have similar EAG responses at higher concentrations of each odorant, which is probably because individual ORN firing rates are known to reach a maximum and plateau [1,25]. These data thus show that the changes in ab3A response between the three different phenotypes result in changes in broader antennal neuronal responses.

### 2.2. Flies with the ab3A-1 and ab3A-2 phenotypes have differing behavioural preferences for ethyl hexanoate

We then proceeded to investigate whether flies with the different ab3A phenotypes exhibit differences in olfactory behaviour responses. We were particularly interested in determining if we could identify behavioural differences between flies with the long and short *Or22* allele. As mentioned, these may show latitudinal clinal distribution in Australia, with high frequencies of the short allele in the north and the long allele fully penetrant in the south. While both the ab3A-1 and ab3A-3 phenotypes are associated with the long allele, in our earlier study of this locus all isogenic lines that we derived from southern populations had the ab3A-1 phenotype. We therefore tested for behavioural differences between the ab3A-1 and ab3A-2 phenotypes. We used ethyl hexanoate as our test odorant because this odorant is not detected by other neurons at lower concentrations [9,11,24], and ab3A-1 and ab3A-2 flies detect this odorant differently at the level of both the individual neuron [10] and the antenna (figure 1).

**Figure 2.**
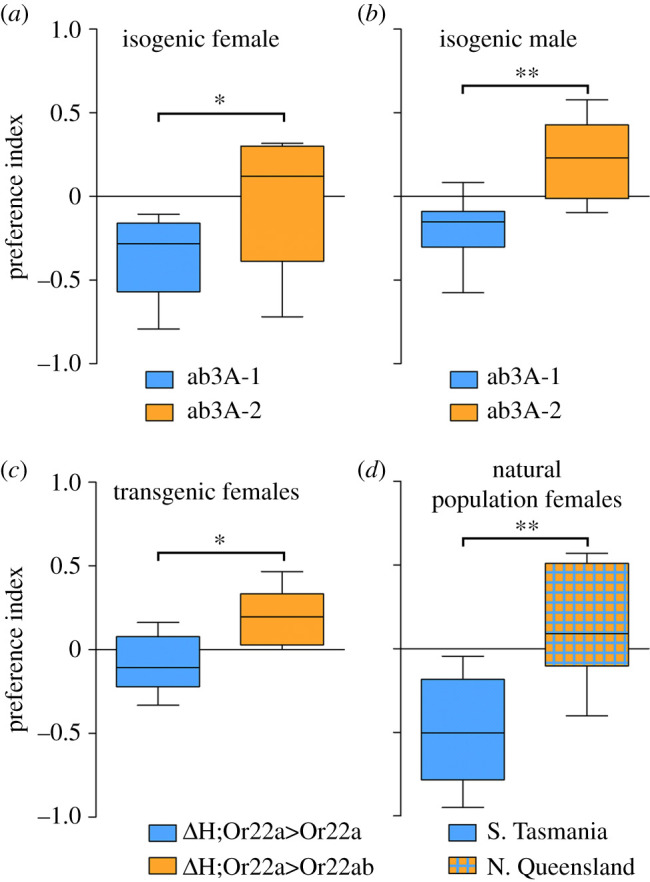
*Or22* genotype correlates with olfactory behavioural response in a two-choice cage assay. (*a,b*) Female and male flies from the ab3A-2 isogenic line (orange) show greater attraction to ethyl hexanoate than the same sex flies from the ab3A-1 isogenic line (blue; Mann–Whitney *U*, *a*: *p* = 0.038, *b*: *p* = 0.002). Preference indices of the individual isogenic lines do not differ significantly between sexes (ab3A-1: *p* = 0.203, ab3A-2: *p* = 0.275). (*c*) Female flies expressing *Or22ab* in the empty neuron (orange) are more attracted to ethyl hexanoate than flies expressing *Or22a* in the empty neuron (blue; *p* = 0.010). (*d*) Flies from a population with only the *Or22a + Or22b* allele (blue) show significantly less attraction to ethyl hexanoate than flies from a population with approximately 75% frequency of the *Or22ab* allele (orange with blue hatching; *p* = 0.003). Data are presented as the median + interquartile ranges, *n* = 8.

To measure olfactory preference behaviour, we used a two-choice cage assay adapted from Faucher *et al*. [26]. Flies were given a choice between two bottles with an attractive odorant added, one of which is dosed with the odorant of interest. A preference index (PI) was calculated from the number of flies in each bottle at the end of the assay. A negative PI indicates flies preferred the control bottle (C), while a positive PI indicates flies preferred the test bottle (T) with the odorant of interest added. We first tested the ethyl hexanoate preferences of flies from the ab3A-1 and ab3A-2 isogenic lines. We assessed the behavioural choices of males and females separately in order to determine if there was an effect of sex on behaviour. For both females (figure 2*a*) and males (figure 2*b*) we found that ab3A-2 flies were significantly more attracted to ethyl hexanoate than ab3A-1 flies (Mann–Whitney *U* of ab3A-1 versus ab3A-2: females *p* = 0.038; males *p* = 0.004). For both isogenic lines we observed no significant difference in preference between males and females (ab3A-1 males versus females: *p* = 0.206; ab3A-2 males versus females: *p* = 0.278). Given this, we used females only for all further experiments.

We note that the isogenic lines contain other differences in their genetic background aside from the genotype at the *Or22* locus, and thus these could potentially contribute to the behavioural differences observed. Therefore, to further determine if variation at the *Or22* locus leads to behavioural differences, we tested the same behavioural preference for ethyl hexanoate in females expressing either *Or22a* (ab3A-1) or *Or22ab* (ab3A-2) transgenes in the ‘empty neuron’ system [8]. We found a significant difference between the preferences of flies expressing *Or22ab* and those expressing *Or22a* (*p* = 0.014), with flies expressing *Or22ab* (ab3A-2) more attracted to ethyl hexanoate than those expressing *Or22a* (ab3A-1; figure 2*c*), as would be expected if the difference observed in the isogenic lines is due to the *Or22* locus. We note that in this experiment the genetic background of the flies is not completely identical, as the background of the chromosome carrying the UAS transgene differs. Thus, while the combination of these two experiments is persuasive, we cannot definitively conclude from either alone that the behavioural differences we observe are solely due to the *Or22* genotype.

We therefore performed a third experiment to further support that *Or22* genotype influences attraction to ethyl hexanoate. In this case we tested attraction to ethyl hexanoate in females from two natural fly populations collected from the north and south of Australia. The population from the north (northern Queensland) was determined to have a 74.9% (±1.6%) frequency of the short allele, while the population from the south (southern Tasmania) only has the long allele present. We found that these two populations have significantly different preferences (Mann–Whitney *U*, *p* = 0.003), with flies from the northern population, with the high frequency of *Or22ab*, showing significantly higher attraction to ethyl hexanoate than flies from the southern population, which is fixed for the long allele (figure 2*d*). The combined data from these three different experiments thus strongly suggest that flies with the *Or22ab* variant are more strongly attracted to ethyl hexanoate than are flies in which *Or22a* determines ab3A response, and thus that genotype at the *Or22* locus is associated with variation in olfactory preference behaviour.

### 2.3. Flies with the ab3A-1 and ab3A-2 phenotypes have different fruit oviposition preferences

Ethyl hexanoate and other odorants detected by the ab3A neuron are known to be released from a variety of fruits, including fruits on which *D. melanogaster* flies are known to oviposit. Along the Australian east coast there are different geographical distributions of fruits grown due to different climates. For example, tropical fruits such as banana are grown in the north, whereas fruits that grow in temperate climates, such as apple, are grown predominantly in the south. We therefore wondered if there are differences between the ab3A phenotypes in preference of fruits for oviposition, which might lead to different phenotypes being favoured in different geographical regions, and might explain the observed cline in allele frequencies along the east coast.

To test for oviposition fruit preference, we designed an assay (adapted from that of Stensmyr *et al*. [27]) that gives females a choice between eight different fruit substrates on which to oviposit, as well as a control plate with no fruit added. Each fruit was homogenized and mixed with 1% agarose dyed blue to reduce any visual or textural influences on oviposition site choice. Based on the proportion of eggs laid on each fruit an oviposition PI was calculated that indicates the preference of the flies to oviposit on that particular fruit. For our eight fruits, we selected half that are ‘northern’ (tropical-growing) fruits (banana, mango, pineapple and pomelo) and half that are ‘southern’ (temperate-growing) fruits (apple, apricot, pear and strawberry). This provided the flies with a wide variety of choice, and also allowed us to determine if flies preferred to oviposit on the northern or southern fruits. We used flies from the ab3A-1 and ab3A-2 isogenic lines in this experiment, as flies expressing *Or22a* and *Or22ab* in the empty neuron have very poor fertility (probably due to other genes within the chromosomal deletion in the empty neuron background strain [28]).

**Figure 3.**
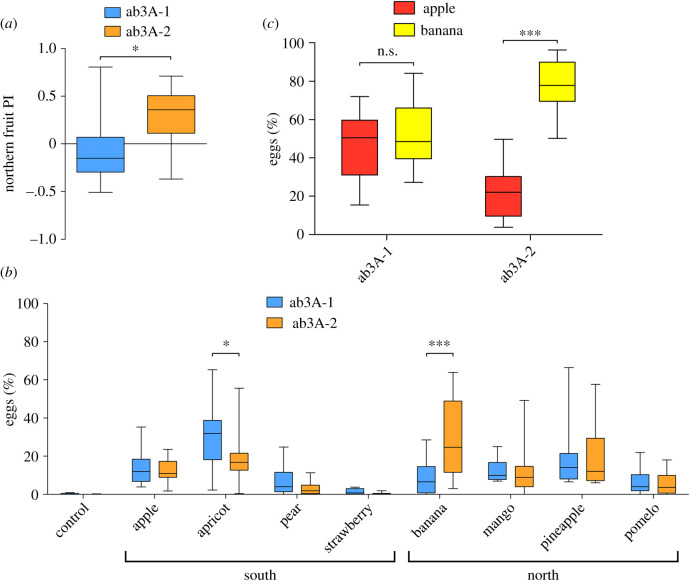
Flies with different ab3A phenotypes show differences in fruit oviposition preference. (*a*) When presented with four fruits typically grown in the south and four typically grown in the north of Australia, isogenic lines with the ab3A-1 (blue) and ab3A-2 (orange) phenotype showed significantly different preferences for ovipositing on northern fruits (Mann–Whitney *U*, *p* = 0.014). Positive index indicates a preference for northern fruits. (*b*) Breaking down the data from (*a*) for the individual fruits, choices of ab3A-1 and ab3A-2 flies differed significantly for apricot and banana (2-way ANOVA with Bonferroni post-tests; apricot *p* < 0.05, banana *p* < 0.001) (*c*) When given a choice between only apple (red) and banana (yellow) as fruit substrates, ab3A-1 flies showed no differences in oviposition site choice, whereas ab3A-2 flies strongly preferred banana (2-way ANOVA with Bonferroni post-tests; ab3A-1 *p* > 0.05, ab3A-2 *p* < 0.001). All data are presented as the median + interquartile range, *n* = 12.

We found that the two isogenic lines showed significantly different preferences for northern and southern fruits, with ab3A-2 flies laying significantly more eggs on northern than southern fruits (figure 3*a*; Mann–Whitney *U*, *p* = 0.014), whereas ab3A-1 flies showed no preference for one group over the other. When these data were broken down to analyse oviposition preference for the individual fruits we found that, while flies from the two isogenic lines showed similar preferences for ovipositing on six of the fruits, ab3A-1 flies had a stronger preference for apricot than did ab3A-2 flies (two-way ANOVA with Bonferroni post-tests, *p* < 0.05), and ab3A-2 flies had a stronger preference for banana than did ab3A-1 flies (*p* < 0.001). The difference in preference for banana was highly significant, and thus to add further evidence to this finding we performed a binary oviposition preference assay where flies had to select between banana and just one other fruit. For this we used apple as a fruit on which both lines laid well (compared to control plates, one-way ANOVA with Dunnett's multiple comparison, ab3A-1 *p* < 0.01, ab3A-2 *p* < 0.05), but for which we had observed no preference difference between ab3A-1 and ab3A-2 flies (two-way ANOVA with Bonferroni post-tests, apple ab3A-1 v ab3A-2, *p* > 0.05). Supporting the initial finding, we found that ab3A-2 flies showed a strong preference for ovipositing on banana (on average laying 77% of their eggs on banana), whereas ab3A-1 flies did not discriminate between apple and banana (figure 3*b*; ab3A-1 *p* > 0.05, ab3A-2 *p* < 0.001). These data therefore suggest that changes in ab3A phenotype correlate with altered oviposition site preference.

## 3. Discussion

Taken together, our data suggest that ab3A response variation due to changes at the *D. melanogaster Or22* locus is associated with differences in two different olfactory-driven behaviours. Our finding that flies carrying the different *Or22* variants show oviposition differences is especially intriguing. Oviposition decisions are crucial to fitness because *Drosophila* flies lay eggs on fermenting fruits where larvae then feed on yeast. It is thus possible that the high frequency of the *Or22ab* allele found in northern Australia reflects a selective advantage in these regions where different fruits are grown. This could be caused by differing sensitivity and behavioural responses to either the fruit-derived odorants themselves, or due to odorants produced by the yeasts and other microbes found in fermenting fruits, which could also differ in these geographical regions.

We note that it is, of course, possible that the differential distribution of *Or22* alleles along the Australian east coast is not indicative of a role of this locus in selection. Instead, as *D. melanogaster* is a human commensal it may have been introduced to Australia multiple times, with different frequencies of the different variants in the founding populations at their northern and southern introduction sites. It is also possible that the *Or22* locus is in linkage disequilibrium with another locus of ecological importance, for example, one involved in heat adaptation. However, given that we find behavioural changes associated with the differences in ab3A response in transgenic flies where only this locus is different, it seems plausible that differences at this locus could lead to clinal distribution of these alleles.

Our work contributes to a growing body of evidence that, among the many classes of ORNs, the ab3A neurons are of particular importance to the ecological specialization of *Drosophila* flies. A recent study showed that a population of forest-dwelling wild *D. melanogaster* in Zimbabwe are seasonal specialists of the highly geographically restricted marula fruit [16]. The authors showed that this fruit releases high levels of the ester ethyl isovalerate, that this is detected by ab3A neurons, and that flies homozygous for *Or22ab* are more sensitive to this odorant than Canton-S flies. They further showed that removal of ab3A neuron function reduced the ability of flies to localize marula fruit, suggesting a key role for this neuron class in this behaviour. There is also significant evidence that the ab3A neuron plays a role in host-specialization in several other *Drosophila* species. *D. sechellia* are specialists of the morinda fruit, and their detection of this fruit is mediated by it releasing methyl hexanoate, which is predominantly detected by ab3A neurons [19]. *D. erecta* are seasonal specialists of the *Pandanus* fruit, and detection of this fruit is predominantly due to the release of 3-methyl-2-butenyl acetate, which is also primarily detected by ab3A neurons [20]. *D. suzukii* is a pest species that, unlike most other *Drosophila* species, prefers fresh rather than rotting fruit. The ab3A neurons of this species have been found to detect β-cyclocitral, a compound to which they are attracted that is released from leaves [21]. This ligand is not detected by the ab3A neurons of *D. melanogaster*, and the authors thus proposed that this change in ligand-specificity of the ab3A neuron is one of the factors involved in the switch of *D. suzukii* from rotting to fresh fruit.

Compared to other ORN classes, the ab3A neuron response profile is unusually variable across different *Drosophila* species [11,12], albeit this analysis of ORN types was not extensive. Paralleling this, in addition to showing genetic variation within *D. melanogaster*, the *Or22* locus is also unique among the *Ors* in exhibiting high levels of genetic variation and extensive levels of copy number variation across species [15]. This suggests that the high level of genetic variability might provide opportunities for functional changes that contribute to the evolution of differences in olfactory-driven behaviours. While naturally occurring changes in the sequence of some other *Or* genes have been implicated in changes in olfactory behaviour [29,30], our data provides one of the first insights into the causative links between Or genetic variation, neuronal response variation and behavioural changes. It will be of great interest in future to determine which *Or22* genetic variants underpin shifts in host-specialization in other *Drosophila* species, and have thus contributed to the ability of these species to evolve and use different ecological niches.

## 4. Material and methods

### 4.1. *Drosophila* stocks

Four mass-bred populations of *D. melanogaster*, originally collected from Bowen, Queensland (19°58′ S), Innisfail, Queensland (17°31′ S), Northern Tasmania (41° S) and Southern Tasmania (43° S), were a kind gift from Carla Sgró, Monash University [31]. The allelic frequencies of the long and short alleles in the northern Queensland population were found by taking 14 samples of 56–92 flies and individually PCR genotyping all flies in the sample using GoTaq (Promega). Primer sequences: Or22ab-F 5′ GCA AGT TTT TTC CCC ACA TT 3′; Or22ab-R 5′ ACC CCA TGA GAA TGA CTT CG 3′; amplicon is 336 bp; Or22a&b-F 5′ GCA GTT TTT CGC AAA GGA AG 3′; Or22a&b-R 5′ AAA GTT TTC CGG GAA TGT CA 3′; amplicon is 639 bp. Isogenic lines were derived as described in Shaw *et al*. [10]. All isogenic lines were maintained on standard wheat-based media at 22°C. To drive expression of olfactory receptor transgenes in the ab3A neuron we used flies carrying *ΔHalo*, a small deletion on the second chromosome that removes the *Or22* locus, combined with an *Or22a*-promoter construct [13]. The *w;ΔHalo/CyO;P[Or22a-Gal4]* and *w;ΔHalo/CyO;P[UAS-Or22a]*, stocks were obtained from John Carlson, Yale University. *P[UAS-Or22ab]* stock is from Shaw *et al*. [10]. All crosses were performed at 25°C.

### 4.2. EAG recordings

Electroantennograms (EAGs) were recorded as per Tom *et al*. [32]. Briefly, a single fly was immobilized and a reference electrode inserted into the eye and a recording electrode placed on the surface of the antenna. Changes in voltage (mV) in response to 1 s stimulations with odorants were amplified using an active probe and a serial-IDAC amplifier (Syntech). EAGs represent the summed activity of a population of ORNs. Odour stimulation was by injecting volatiles from 5 ml syringes into an airstream blown over the preparation. All odorants were at highest available purity (greater than 98%, Sigma-Aldrich) and dissolved in paraffin oil to reach the specified dilution.

### 4.3. Two-choice cage assay

The two-choice cage assay was adapted from Faucher *et al*. [26]. A 305 × 305 × 305 mm cage was fitted with two 66 mm funnels placed 130 mm apart leading into 250 ml glass bottle traps. Bottles were filled with 40 ml solutions of 50% (vol/vol) apple cider vinegar (Cornwall's) to attract flies. The test odorant was added to one of the traps (10^−4^ vol/vol for ethyl hexanoate). When testing isogenic lines and natural populations one hundred flies were starved on 1% agarose for 24 h before being released into the cage. When testing the flies expressing either *Or22a* or *Or22ab* in empty ab3A neurons between 50 and 80 flies were tested, due to the poor health of these flies. The cage was surrounded by an open-top black box to minimize directional visual stimuli and placed in a fume hood to provide ventilation. The assay was run for 17 h under natural light conditions, such that the dawn and dusk activity periods were captured. The side that contained the test odorant was alternated between every repetition. A PI was calculated as (*T* − *C*)/(*T* + *C*) where *T* is the number of flies in the test odorant-containing bottle and *C* is the total number of flies in the control bottle. The preference indices were then compared for significance using a Mann–Whitney *U*-test.

### 4.4. Oviposition behaviour assay

The oviposition assay was adapted from Stensmyr *et al*. [27]. We tested eight fruits representing those commonly grown in tropical northern Australia (banana, mango, pineapple and pomelo) and in temperate southern Australia (apple, apricot, pear and strawberry). Fruits were purchased from a fruit and vegetable market, aged for 3 days at room temperature and then homogenized and frozen. One gram of thawed homogenized fruit was added to the lid of a 35 mm tissue culture plate (Sarstedt) with two drops of blue food dye and 4 ml of 1% agarose minimizing both visual and textural differences. Once cooled and set, the plates were placed into a 235 × 235 × 120 mm plastic box with a damp sponge for humidity, a lid with holes for ventilation and left for 24 h at room temperature (22°C) before flies were added. Nine plates were placed in a 3 × 3 grid pattern; one for each of the eight fruits in a pseudorandomized distribution and a control plate containing water in the middle position. When only apple and banana were tested the two fruits were distributed in a regular pattern over the eight plates, with the fruit in the corner plate alternating and the control plate remaining the same. Virgin females and males were kept separately for three days and then crossed. After 2 days 40 fertilized females were placed in a small cage with a food plate coated with yeast paste to induce laying. After 2 days laying on food plates, 30 females were gently sedated by cooling and released in the box. The assay was run for 24 h and the number of eggs laid on each plate was counted to calculate the proportion of eggs laid on each fruit out of the total.

## Data accessibility

All data are available within the article or supporting electronic material, including the statistical analysis supporting this article as electronic supplementary material, table S1. The data are provided in electronic supplementary material [33].

## References

[1] de Bruyne M, Foster K, Carlson JR. 2001 Odor coding in the *Drosophila* antenna. Neuron **30**, 537-552. (10.1016/S0896-6273(01)00289-6) 11395013

[2] de Bruyne M, Warr CG. 2006 Molecular and cellular organization of insect chemosensory neurons. BioEssays: News Rev. Mol. Cell. Dev. Biol. **28**, 23-34. (10.1002/bies.20338) 16369946

[3] Haverkamp A, Hansson BS, Knaden M. 2018 Combinatorial codes and labeled lines: how insects use olfactory cues to find and judge food, mates, and oviposition sites in complex environments. Front. Physiol. **9**, 49. (10.3389/fphys.2018.00049) 29449815 PMC5799900

[4] Clyne PJ, Warr CG, Freeman MR, Lessing D, Kim J, Carlson JR. 1999 A novel family of divergent seven-transmembrane proteins: candidate odorant receptors in *Drosophila*. Neuron **22**, 327-338. (10.1016/S0896-6273(00)81093-4) 10069338

[5] Benton R, Vannice KS, Gomez-Diaz C, Vosshall LB. 2009 Variant ionotropic glutamate receptors as chemosensory receptors in *Drosophila*. Cell **136**, 149-162. (10.1016/j.cell.2008.12.001) 19135896 PMC2709536

[6] Gomez-Diaz C, Martin F, Garcia-Fernandez JM, Alcorta E. 2018 The two main olfactory receptor families in *Drosophila*, ORs and IRs: a comparative approach. Front. Cellular Neurosci. **12**, 253. (10.3389/fncel.2018.00253) PMC6125307 30214396

[7] Yan H, Jafari S, Pask G, Zhou X, Reinberg D, Desplan C. 2020 Evolution, developmental expression and function of odorant receptors in insects. J. Exp. Biol. **223**, jeb208215. (10.1242/jeb.208215) 32034042 PMC7790194

[8] Hallem EA, Ho MG, Carlson JR. 2004 The molecular basis of odor coding in the *Drosophila* antenna. Cell **117**, 965-979. (10.1016/j.cell.2004.05.012) 15210116

[9] Hallem EA, Carlson JR. 2006 Coding of odors by a receptor repertoire. Cell **125**, 143-160. (10.1016/j.cell.2006.01.050) 16615896

[10] Shaw KH, Johnson TK, Anderson A, de Bruyne M, Warr CG. 2019 Molecular and functional evolution at the odorant receptor *Or22* locus in *Drosophila melanogaster*. Mol. Biol. Evol. **36**, 919-929. (10.1093/molbev/msz018) 30768139 PMC6502086

[11] Stensmyr MC, Dekker T, Hansson BS. 2003 Evolution of the olfactory code in the *Drosophila melanogaster* subgroup. Proc. R. Soc. B **270**, 2333-2340. (10.1098/rspb.2003.2512) PMC1691514 14667348

[12] de Bruyne M, Smart R, Zammit E, Warr CG. 2010 Functional and molecular evolution of olfactory neurons and receptors for aliphatic esters across the *Drosophila* genus. J. Comp. Physiol. A Neuroethol. Sensory Neural Behav. Physiol. **196**, 97-109. (10.1007/s00359-009-0496-6) 20033746

[13] Dobritsa AA, van der Goes van Naters W, Warr CG, Steinbrecht RA, Carlson JR. 2003 Integrating the molecular and cellular basis of odor coding in the *Drosophila* antenna. Neuron **37**, 827-841. (10.1016/S0896-6273(03)00094-1) 12628173

[14] Turner TL, Levine MT, Eckert ML, Begun DJ. 2008 Genomic analysis of adaptive differentiation in *Drosophila melanogaster*. Genetics **179**, 455-473. (10.1534/genetics.107.083659) 18493064 PMC2390623

[15] Aguadé M. 2009 Nucleotide and copy-number polymorphism at the odorant receptor genes *Or22a* and *Or22b* in *Drosophila melanogaster*. Mol. Biol. Evol. **26**, 61-70. (10.1093/molbev/msn227) 18922763

[16] Mansourian S, Enjin A, Jirle EV, Ramesh V, Rehermann G, Becher PG, Pool JE. 2018 Wild African *Drosophila melanogaster* are seasonal specialists on marula fruit. Curr. Biol. **28**, 3960-3968. (10.1016/j.cub.2018.10.033) 30528579 PMC7065024

[17] Hale LR, Singh RS. 1991 A comprehensive study of genic variation in natural populations of *Drosophila* *melanogaster*. IV. Mitochondrial DNA variation and the role of history vs. selection in the genetic structure of geographic populations. Genetics **129**, 103-117. (10.1093/genetics/129.1.103) 1682210 PMC1204559

[18] Singh RS, Rhomberg LR. 1987 A comprehensive study of genic variation in natural populations of *Drosophila* *melanogaster*. II. Estimates of heterozygosity and patterns of geographic differentiation. Genetics **117**, 255-271. (10.1093/genetics/117.2.255) 17246403 PMC1203202

[19] Dekker T, Ibba I, Siju KP, Stensmyr MC, Hansson BS. 2006 Olfactory shifts parallel superspecialism for toxic fruit in *Drosophila melanogaster* sibling, *D. sechellia*. Curr. Biol. **16**, 101-109. (10.1016/j.cub.2005.11.075) 16401429

[20] Linz J, Baschwitz A, Strutz A, Dweck HKM, Sachse S, Hansson BS, Stensmyr MC. 2013 Host plant-driven sensory specialization in *Drosophila erecta*. Proc. R. Soc. B **280**, 20130626. (10.1098/rspb.2013.0626) PMC3652467 23595274

[21] Keesey IW, Knaden M, Hansson BS. 2015 Olfactory specialization in *Drosophila suzukii* supports an ecological shift in host preference from rotten to fresh fruit. J. Chem. Ecol. **41**, 121-128. (10.1007/s10886-015-0544-3) 25618323 PMC4351439

[22] Andersson MN, Schlyter F, Hill SR, Dekker T. 2012 What reaches the antenna? How to calibrate odor flux and ligand–receptor affinities. Chem. Senses **37**, 403-420. (10.1093/chemse/bjs009) 22362868

[23] Ayer RK, Carlson J. 1991 *acj6*: a gene affecting olfactory physiology and behavior in *Drosophila*. Proc. Natl Acad. Sci. USA **88**, 5467. (10.1073/pnas.88.12.5467) 1905022 PMC51894

[24] Stensmyr MC, Giordano E, Balloi A, Angioy A-M, Hansson BS. 2003 Novel natural ligands for *Drosophila* olfactory receptor neurones. J. Exp. Biol. **206**, 715. (10.1242/jeb.00143) 12517989

[25] de Bruyne M, Clyne PJ, Carlson JR. 1999 Odor coding in a model olfactory organ: the *Drosophila* maxillary palp. J. Neurosci. **19**, 4520-4532. (10.1523/JNEUROSCI.19-11-04520.1999) 10341252 PMC6782632

[26] Faucher CP, Hilker M, de Bruyne M. 2013 Interactions of carbon dioxide and food odours in *Drosophila*: olfactory hedonics and sensory neuron properties. PLoS ONE **8**, e56361. (10.1371/journal.pone.0056361) 23457557 PMC3574157

[27] Stensmyr MC et al. 2012 A conserved dedicated olfactory circuit for detecting harmful microbes in *Drosophila*. Cell **151**, 1345-1357. (10.1016/j.cell.2012.09.046) 23217715

[28] Gross SP, Guo Y, Martinez JE, Welte MA. 2003 A determinant for directionality of organelle transport in *Drosophila* embryos. Curr. Biol. **13**, 1660-1668. (10.1016/j.cub.2003.08.032) 14521831

[29] Richgels PK, Rollmann SM. 2012 Genetic variation in odorant receptors contributes to variation in olfactory behavior in a natural population of *Drosophila melanogaster*. Chem. Senses **37**, 229-240. (10.1093/chemse/bjr097) 22038943 PMC3278676

[30] Rollmann SM, Wang P, Date P, West SA, Mackay TFC, Anholt RRH. 2010 Odorant receptor polymorphisms and natural variation in olfactory behavior in *Drosophila melanogaster*. Genetics **186**, 687-697. (10.1534/genetics.110.119446) 20628035 PMC2954467

[31] Sgrò CM, Overgaard J, Kristensen TN, Mitchell KA, Cockerell FE, Hoffmann AA. 2010 A comprehensive assessment of geographic variation in heat tolerance and hardening capacity in populations of *Drosophila melanogaster* from eastern Australia. J. Evol. Biol. **23**, 2484-2493. (10.1111/j.1420-9101.2010.02110.x) 20874849

[32] Tom W, de Bruyne M, Haehnel M, Carlson JR, Ray A. 2011 Disruption of olfactory receptor neuron patterning in Scutoid mutant *Drosophila*. Mol Cell Neurosci. **46**, 252-261. (10.1016/j.mcn.2010.09.008) 20875862 PMC3019251

[33] Shaw KH, Dent CI, Johnson TK, Anderson A, de Bruyne M, Warr CG. 2021 Natural variation at the *Drosophila melanogaster**Or22* odorant receptor locus is associated with changes in olfactory behaviour. *Figshare*. 10.1098/rsob.210158 PMC8478520 34582710

